# Assessing Factors in Time to Diagnosis for Head-and-Neck Lymphoma

**DOI:** 10.7759/cureus.93596

**Published:** 2025-09-30

**Authors:** Rishi Kondapaneni, Eric Y Du, Carson K Gates, Vipul K Bhanderi, Patrick T Tassone

**Affiliations:** 1 Department of Otolaryngology, Head and Neck Surgery, University of Missouri, Columbia, USA; 2 Department of Hematology and Oncology, University of Missouri, Columbia, USA

**Keywords:** fine-needle aspiration, fine-needle aspiration accuracy, head and neck lymphoma, otolaryngology head and neck, time to diagnosis

## Abstract

Objectives

This study aimed to identify and evaluate factors influencing time to diagnosis (TTD) in head-and-neck (H&N) lymphoma, including fine-needle biopsy (FNA), and identify points of improvement in the management practice of H&N lymphoma.

Methods

This retrospective cohort study was conducted at University of Missouri, a tertiary care health system in Columbia, Missouri. Electronical medical records were reviewed to identify all patients who presented to otolaryngology and were subsequently diagnosed with lymphoma in the H&N region. Demographic and clinical factors influencing TTD, defined as time from initial clinical visit to final pathologic result, were evaluated using Cox proportional hazards multivariable regression modelling and visualized using Kaplan-Meier survival curves.

Results

Our study sample had 137 patients, most of whom presented with the primary symptom of neck mass/lymphadenopathy (81.0%). A personal history of lymphoma (HR 1.81 (1.03, 3.18); p = 0.04) and initial presenting symptoms of other than lymphadenopathy or neck mass (e.g., imaging findings, HR 2.12 (1.26, 3.59); p < 0.01) were factors associated with shorter TTD, whereas older age (HR 0.98 (0.97, 0.99) per year; p < 0.01) portends longer TTD. While FNA completed by ENT had a higher proportion of above median TTD (64.5% vs 40.0%; p < 0.01) on univariate analysis, this finding was not presenting after controlling for covariables (HR 0.93 (0.63, 1.39); p = 0.73).

Conclusion

We identified several factors associated with TTD in H&D lymphoma. FNA for lesions subsequently diagnosed as lymphoma does not clearly lengthen TTD, after controlling for confounders.

## Introduction

Time to diagnosis (TTD) is an important prognostic factor for many aggressive malignancies, with the literature commonly reporting a negative association between TTD and prognosis [1]. However, there exists little data on TTD in head-and-neck (H&N) presentations of lymphoma. Atypical presentations of H&N lymphoma (i.e., salivary gland involvement, lack of discrete lymphadenopathy, etc.) may challenge clinical recognition, thus affecting TTD and subsequent treatment initiation [2].

Previous studies have outlined the natural progression of patient workup in cases of lymphoma, identifying time intervals between pivotal points in patient care (i.e., primary care provider contact to specialist referral, hospital appointment to diagnosis, etc.) [3]. While lymphadenopathy and “B-symptoms” in an older adult may trigger rapid evaluation, certain characteristics such as a recent febrile illness or transient lymph node swelling may lead providers to favor a benign process [3,4]. Thus, the delays in diagnostic workup may compound into a significantly longer TTD. In addition, the vast variability in presenting symptoms may lead to poorer medical management and diagnostic delay [2].

Currently, much of the literature investigating TTD intervals exists for lymphoma as a whole (rather than isolated to the H&N region) or to mucosal H&N cancers, including squamous cell carcinoma [5-11]. The factors associated with TTD in cases of H&N lymphoma are poorly understood. The vast variability in clinical presentations often prompts a range of clinical approaches, including fine-needle aspiration (FNA), that may insidiously delay the process for definitive diagnosis, ultimately affecting TTD, subsequent treatment, and potentially patient outcomes [2,5].

Specifically, FNA is a point of contention in the workup of H&N lymphoma. It is considered a first-line diagnostic study in the workup of H&N masses because it is cost-effective and minimally invasive [12]. However, it does not maintain tissue architecture, and other literature contends that FNA for the diagnosis of lymphoma is not useful and may even misguide treatment decisions [13,14]. Other diagnostic procedures include core needle biopsy and excisional biopsy, the latter of which is considered the gold standard for diagnosis of lymphoma [12]. Because the results of a fine needle aspiration must be sent for analysis by a pathologist, there is a concern that FNA may increase the TTD in patients with lymphoma without being a useful diagnostic tool.

This study aims to identify factors that influence TTD in patients who present to otolaryngology and are subsequently diagnosed with H&N lymphoma. Specifically, we look to evaluate demographic and medical characteristics, including if the patient received an FNA biopsy prior to definitive excisional biopsy for diagnosis, to assess if there are areas that need to be addressed in care and identify patients at risk for longer TTD.

## Materials and methods

Study design and sample

This is a retrospective cohort study of adult and pediatric patients ranging from age six to 86 who presented to the Otolaryngology Clinic at the University of Missouri, a university hospital in Columbia, Missouri, and were subsequently diagnosed with pathologically confirmed lymphoma with excisional biopsy and treated between January 1, 2004, and January 1, 2024. This research was approved by the University of Missouri Institutional Review Board, Columbia, MO, USA (IRB 2099015) with full HIPAA (Health Insurance Portability and Accountability Act) waiver granted. Patients were identified and included in the study by a diagnosis of lymphoma and contact with an otolaryngology specialist. Inclusion criteria include diagnosed lymphoma of the H&N region, and diagnostic evaluation performed by an otolaryngology specialist. Those with a diagnosis of lymphoma in other body areas, no contact with Otolaryngology, or diagnostic workup performed by a non-otolaryngology specialty (e.g., interventional radiology) were excluded from the study. Patients with no discrete diagnosis date or who had incomplete records impeding complete data collection were excluded.

Measures and covariates

The electronic medical records of each identified patient were reviewed; specifically, the clinical documentation from and the dates of otolaryngology, hematology oncology, or radiation oncology outpatient visits, otolaryngology surgical operative reports, surgical pathology reports, and radiographic imaging studies were recorded and subsequently stored in a REDCap database. Specifically, patient sociodemographic characteristics, including age (years), sex (male, female), and smoking status (current, former, nonsmoker); pertinent clinical information, including time between otolaryngology visit and excisional biopsy, final pathologic diagnosis, and lymphoma treatment initiation; details of evaluation, including imaging and any procedural biopsy aside from surgical excisional biopsy (FNA, core needle biopsy); and lymphoma details, including presenting clinical symptom (lymphadenopathy/neck mass, imaging findings, others), type and subtype of lymphoma, lymphoma stage, and any personal history of lymphoma. The primary dependent variable was time from initial otolaryngology clinical visit to final pathologic result via excisional biopsy, in days; the secondary dependent variable is time from initial otolaryngology clinical visit to initiation of treatment for lymphoma, in days.

Statistical analysis

Descriptive statistics were used to analyze characteristics across the entire patient sample and stratified by time from ENT consultation to final pathology result, using a cutoff of below or equal to the median time of three weeks versus >3 weeks. Covariable distributions were compared using one-way analysis of variance for continuous variables and the Pearson χ² test for categorical variables.

To evaluate factors associated with time in days from ENT consultation to key clinical milestones, Cox proportional hazards regression was employed for time-to-event analysis. Two separate models were constructed: Model 1 evaluated factors associated with time from ENT consultation to pathology result for primary analysis, and Model 2 assessed time from ENT consultation to treatment initiation as a secondary analysis. Predictor covariates included age, sex, history of lymphoma, lymphoma type (reference: Hodgkin lymphoma), disease stage (reference: I/II), presenting symptom (reference: lymphadenopathy/neck mass), and whether a fine-needle aspiration (FNA) biopsy was performed by ENT. Hazard ratios (HRs) with corresponding 95% confidence intervals (CIs) and p-values are reported. Kaplan-Meier survival curves were generated to illustrate time from ENT consultation to pathology result. All statistical analysis and figure creation were conducted using Stata statistical software version 17.0 (Stata Corporation, College Station, TX). All statistical analyses were two-tailed, and the alpha threshold for statistical significance was a two-sided p-value of 0.05.

## Results

Descriptive statistics

Of the 137 patients with confirmed lymphoma on final pathology from excisional biopsy, the majority were male (62.8%), nonsmokers (55.9%) of mean (SD) age of 53.1 (17.3) years at presentation (Table 1). Most of the patients presented to ENT clinic with primary presenting symptom of neck mass or lymphadenopathy (81.0%). A minority (19.7%) endorsed B symptoms such as weight loss or night sweats. Only 20 patients (14.6%) presented with a prior history of lymphoma.

**Table 1:** Descriptive statistics comparing patient characteristics by time from ENT consultation to pathology result, stratified by the median cutoff of >3 weeks (cohorts are <3 weeks vs >= 3 weeks). Data are presented as mean (SD) for continuous measures and % (n) for categorical measures. Comparisons between groups were conducted using appropriate statistical tests (e.g., chi-square or Fisher’s exact test for categorical variables; one way ANOVA for continuous variables) Oto-HNS: otolaryngology – head and neck surgery

Variable	Total (n = 137)	≤3 weeks (n = 75)	>3 weeks (n = 62)	p-value
Age at presentation	53.1 (17.3)	49.9 (16.8)	57.0 (17.1)	0.01
Sex				0.98
Male	62.8% (86)	62.7% (47)	62.9% (39)	
Female	37.2% (51)	37.3% (28)	37.1% (23)	
Smoking status				0.10
Nonsmoker	55.9% (76)	62.7% (47)	47.5% (29)	
Current smoker	28.7% (39)	21.3% (16)	37.7% (23)	
Former smoker (quit >2 years ago)	15.4% (21)	16.0% (12)	14.8% (9)	
Presenting symptom				0.19
Lymphadenopathy / neck mass	81.0% (111)	76.0% (57)	87.1% (54)	
Imaging findings	9.5% (13)	13.3% (10)	4.8% (3)	
Other symptoms	9.5% (13)	10.7% (8)	8.1% (5)	
History of lymphoma				0.01
No	85.4% (117)	78.7% (59)	93.5% (58)	
Yes	14.6% (20)	21.3% (16)	6.5% (4)	
Type of lymphoma				0.12
Hodgkin lymphoma	21.2% (29)	26.7% (20)	14.5% (9)	
B-cell non-Hodgkin lymphoma	72.3% (99)	69.3% (52)	75.8% (47)	
T-cell non-Hodgkin lymphoma	6.6% (9)	4.0% (3)	9.7% (6)	
Lymphoma stage				0.80
I/II	54.4% (74)	55.4% (41)	53.2% (33)	
III/IV	45.6% (62)	44.6% (33)	46.8% (29)	
FNA biopsy				
Performed prior to Oto-HNS visit	10.2% (14)	12.0% (9)	8.1% (5)	0.45
Performed by Oto-HNS	51.1% (70)	40.0% (30)	64.5% (40)	<0.01
Ordered by Oto-HNS	2.2% (3)	2.7% (2)	1.6% (1)	0.67

Prior to the initial ENT visit, 77 patients (56.2%) and 18 patients (13.1%) had a prior computed tomography (CT) scan/ultrasound of the neck completed, respectively, and 30 patients had CT scan of the neck ordered subsequent to the visit. Fourteen patients (10.2%) had an FNA completed prior to initial ENT visit, which an additional 70 patients (51.1%) having an FNA completed by the ENT during the visit (of which three patients had an FNA prior to the visit), and an additional three FNAs were ordered by OTO to be completed by interventional radiology. Of the 84 initial FNA results, 58 (69.1%) suggested lymphoma, 18 (21.4%) were nondiagnostic, and eight (9.5%) suggested a different diagnosis.

Following final pathologic diagnosis by excisional biopsy, the majority of patients were diagnosed with a B-cell non-Hodgkin lymphoma (n = 99, 72.3%) at approximately equal incidence of early versus late stage (54.4% vs. 45.6%, respectively). Following treatment, 106 (79.7%) patients had complete remission and 19 (14.3%) patients developed recurrence.

Length of TTD and treatment

In the entire sample, it took a median (interquartile range) of 13 (8-24) days from initial ENT consultation to excisional biopsy, 21 (15-32) days to final pathologic diagnosis, and 43 (28-63) days to treatment initiation. In univariate analysis, the 62 patients who took above the median time interval of three weeks from ENT visit to final pathologic result were compared to the 75 patients whose time interval was equal to or less than the median. Age at presentation (mean (SD) of 57.0 (17.1) vs. 49.9 (16.8) years, respectively; p = 0.01) and FNA biopsy performed by ENT (64.5% vs. 40.0%; p < 0.01) were associated with above median time to pathologic diagnosis (Table 1). By contrast, a personal history of lymphoma was associated with below median time to pathologic diagnosis (21.3% vs. 6.5%; p = 0.01). Patient sex, smoking status, presenting symptom, and type and stage of lymphoma did not demonstrate statistically significant differences on univariable analysis.

**Figure 1:**
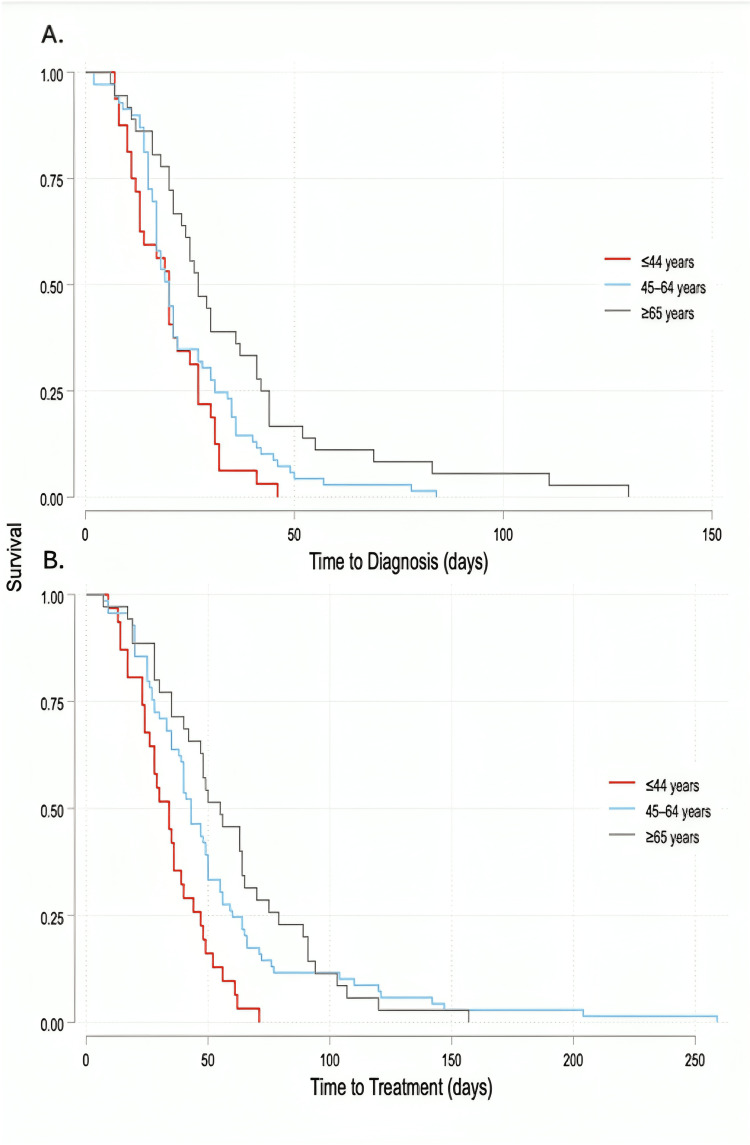
Kaplan-Meier survival curves illustrating time from A. ENT consultation to pathology result and B. ENT consultation to treatment initiation, stratified by age quartiles. Differences in time to pathology across age groups were assessed using the log-rank test. The log-rank test indicates a statistically significant difference in time to event (e.g., time to pathology result and treatment initiation) between the two groups (p < 0.01) on both analyses. Therefore, survival distributions are not equal.

On Cox-proportional hazard multivariable time to event analysis of factors associated with time to pathologic result (Table 2), controlling for covariates as listed previously, factors associated with earlier time to pathologic result (i.e., greater hazard of pathologic result event) include a personal history of lymphoma (hazard ratio (95% CI): 1.81 (1.03, 3.18); p =0.04) and initial presenting symptom of incidental cross-sectional imaging findings/changes or other symptoms as compared to lymphadenopathy or neck mass (HR 2.12 (1.26, 3.59); p < 0.01). Factors associated with later time to pathologic result include older age (HR 0.98 (0.97, 0.99) per year; p < 0.01). Representative Kaplan-Meier curve of time to pathologic diagnosis and time to treatment initiation, categorized by age quartiles, are displayed in Figure 1. T-cell type of non-Hodgkin lymphoma as compared to Hodgkin lymphoma was associated with later TTD, but this did not reach the alpha threshold at 0.05 (HR 0.45 (0.20, 1.04); p = 0.06). On secondary analysis analyzing time from initial ENT visit to treatment initiation had congruent results with the exception of a personal history of lymphoma, which did not demonstrate earlier time to treatment initiation (HR 1.16 (0.67, 2.00); p = 0.61). Overall, in either analysis, FNA biopsy by ENT was not associated with earlier TTD or treatment after controlling for other covariables (HR 0.93 (0.63, 1.39), HR 1.14 (0.74, 1.75); p = 0.73 and 0.55, respectively).

**Table 2:** Cox proportional hazards regression results evaluating factors associated with the outcome of interest. Model 1 includes the initial adjusted analysis; Model 2 represents a secondary model adjusting for additional or alternative covariates. Hazard ratios (HRs) are presented with corresponding 95% confidence intervals (CI) and p-values. Reference categories are indicated where applicable. * p < 0.10, ** p < 0.05. Oto-HNS = otolaryngology – head and neck surgery

Variable	Time from ENT to pathology		Time from ENT to treatment	
	Hazard ratio (95% CI)	p-value	Hazard ratio (95% CI)	p-value
Age (continuous, per year)	0.98 (0.97, 0.99)**	<0.01**	0.98 (0.97, 0.99)**	<0.01**
Sex				
Female	ref.		ref.	
Male	1.13 (0.78, 1.62)	0.53	0.89 (0.61, 1.30)	0.54
History of lymphoma				
No	ref.		ref.	
Yes	1.81 (1.03, 3.18)**	0.04**	1.16 (0.67, 2.00)	0.61
Lymphoma type				
Hodgkin lymphoma	ref.		ref.	
B-cell non-Hodgkin lymphoma	0.75 (0.48, 1.18)	0.21	1.11 (0.70, 1.76)	0.66
T-cell non-Hodgkin lymphoma	0.45 (0.20, 1.04)*	0.06*	0.47 (0.20, 1.13)*	0.09*
Stage				
I/II	ref.		ref.	
III/IV	0.83 (0.58, 1.17)	0.29	0.84 (0.58, 1.20)	0.33
Presenting symptom				
Lymphadenopathy / neck mass	ref.		ref.	
Imaging findings / other symptoms	2.12 (1.26, 3.59)**	<0.01**	1.72 (1.01, 2.92)**	0.04**
FNA biopsy by Oto-HNS				
No	ref.		ref.	
Yes	0.93 (0.63, 1.39)	0.73	1.14 (0.74, 1.75)	0.55

## Discussion

Optimizing TTD intervals is important in many disease processes, but especially malignancy. Significant barriers to timely workup include delayed time in referral to a specialist, issues in obtaining a timely appointment with said specialist, and delayed treatment post-referral [3]. In addition, in a study evaluating TTD for H&N cancers as a whole (i.e., not limited to lymphoma), it was found that TTD varied significantly based on a plethora of patient factors (i.e., sex, smoking status, regular healthcare visits) [7], highlighting the variability in patient characteristics and presentations.

Delayed diagnosis is associated with later-stage disease and potentially poor patient outcomes [8], including shortened progression-free survival in patients with diffuse large B-cell lymphoma presenting in any site [15]. While the literature regarding TTD and prognosis of H&N lymphoma is sparse, multiple studies have previously reported a positive association between faster TTD and better prognosis in patients with non-lymphomatous H&N cancer [10,11,16].

Thus, the present study aimed to identify and evaluate the factors associated with TTD for H&N lymphoma. Our results indicate that there are identifiable variables in both patient characteristics (i.e., age, presenting symptom) and diagnostic workup (i.e., FNA, imaging) that are predictors of a patient’s TTD. Thus, providers should be aware of these variables to optimize efficiency during patient workup for H&N lymphoma.

Factors affecting diagnostic timelines

Upon univariate analysis, patients who had an FNA biopsy performed by ENT had a longer TTD, which is expected given the added diagnostic step that also requires pathological analysis. However, after controlling for covariates, FNA by ENT was not associated with earlier or later TTD. This may reflect confounding factors such as age, presenting symptoms/clinical suspicion, and provider-specific practice that cloud the relationship between FNA and TTD. The role of FNA in the diagnosis of lymphoma remains under debate; supporters argue that it is a cost-effective, minimally invasive, and rapid tool for workup while dissenters assert that the test may be inaccurate, insufficient, cause unnecessary delays in some patients, and have a high false-negative rate [12-14,17]. Our findings support FNA as a reasonable tool for diagnostic workup as it did not prolong TTD or time to treatment in cases of H&N lymphoma. However, limitations remain. In this study alone, only 58 out of 84 FNAs completed prior to ENT visit or by ENT correctly suggested lymphoma: a false-negative rate of 31%. Despite this high false-negative rate, FNA remained a commonly used initial diagnostic tool in this cohort. While FNA may not statistically delay diagnosis, its high false-negative rate and limited sensitivity may still play a role in clinical management.

A prior history of lymphoma was associated with faster TTD in multivariate analysis in this study, as providers likely had a higher index of suspicion for malignancy in this population. However, it did not contribute to a faster time to treatment initiation. Current literature indicates that post-treatment surveillance protocols are well-established and that patients are generally well-informed about recognizing relapse symptoms [18], which may facilitate more timely care delivery. In this study, there was an approximate five-day difference in TTD between patients with past history of lymphoma and those without one (22.0 days vs. 27.1 days); an approximate four-day difference is reflected in the time to treatment (47.4 days vs. 51.2 days). Thus, it is possible that while those with a history of lymphoma are truly diagnosed faster, the interval between diagnosis and treatment itself is not significantly impacted. This interval may be affected by a variety of factors, such as establishment of care, Hematology/Oncology appointment referral/scheduling, port placement, chemotherapy education, etc. Thus, once the diagnosis of lymphoma (or diagnosis of recurrence) is established, our research found that a history of lymphoma is not furthermore significant.

Characteristic imaging findings/non-lymphadenopathy symptoms were associated with a faster TTD and time to treatment as compared to lymphadenopathy on multivariate analysis. While 81% (n = 111) presented with lymphadenopathy, those with incidental imaging findings (n = 13) and “other symptoms” (n = 13) were diagnosed and treated faster. Imaging findings and atypical symptoms likely prompted a quicker workup than lymphadenopathy alone, which has a broad differential. Previous literature supports that CT and MRI scans are useful in expediting cancer workup, although it may be difficult to discern lymphoma from other malignancies with imaging alone without profound lymphadenopathy [19].

Older age was associated with a longer TTD and time to treatment on multivariate analysis and Kaplan-Meier curves. Our study identifies that as age increases, the time from ENT to pathology and ENT to treatment also increases; thus, there must be a factor present after contact with ENT that correlates to increased TTD and time to treatment as related to increased age. This finding does not agree with current literature; one study found that age does not play a significant role in TTD or time to treatment of hematological malignancies including lymphoma [20], while another found that TTD intervals in patients with various types of cancer is more dependent on the presence of characteristic symptoms with no reported effect of age [21]. However, the risk of aggressive disease in the elderly exists; the literature suggests that older patients with lymphoma had a higher frequency of B-symptoms, palpable lymphadenopathy, and lab findings suggestive of aggressive disease [6]. Possible mechanisms underlying this finding include the presence of comorbidites that confound clinical decision-making, delayed referral pathways from primary care to Otolaryngology, or even decreased access to healthcare services in the elderly population. Regardless of the mechanism, this finding underscores the need for prompt evaluation in the older adult population.

Overall, this is a 137-patient study spanning 20 years of care at the University of Missouri Health system. Strengths of the study include the large time span of pathologically confirmed cases, the focused evaluation of FNA impact on TTD, and a Cox proportional hazards model to maximize internal validity. Limitations of the study are the single-center nature of this research limiting the generalizability of results and the possibility of selection bias as our protocol may have excluded patients diagnosed with head-and-neck lymphoma through specialties other than Otolaryngology. Other specialty services may have different management practices (i.e., greater reliance on FNA, rapid employment of imaging) which may lead to the selection bias noted previously. In addition, the findings noted herein do not necessarily have a direct correlation with patient outcomes, but rather only with TTD. A multi-center study incorporating routes of diagnosis through various specialties would serve well to mitigate potential system-specific factors and allow a more objective view of TTD in H&N lymphoma as a whole.

## Conclusions

This single-center retrospective cohort study found that patient history of lymphoma and imaging findings were associated with a faster TTD, increased age is associated with longer TTD, and FNA does not significantly increase TTD for patients with H&N lymphoma. The finding that increased age may prolong TTD deserves further exploration given that prior studies suggest increased disease severity and burden in this population. Importantly, this study provides evidence for the use of FNA in the diagnostic workup of H&N lymphoma. Further research via multi-center studies are needed to mitigate the effect of provider- and system-specific practices that lead to variability in TTD/treatment across institutions.
